# Tuning of the Electronic and Magnetic Properties of GaN Monolayers via Doping with Lanthanide Atoms and by Applying Biaxial Strain

**DOI:** 10.3390/nano15171331

**Published:** 2025-08-29

**Authors:** Xue Wen, Bocheng Lei, Lili Zhang, Haiming Lu

**Affiliations:** 1 Xinjiang Laboratory of Phase Transitions and Microstructures in Condensed Matters, College of Physical Science and Technology, Yili Normal University, Yining 835000, China; 19899036782wx@sina.com (X.W.); leibocheng@ylnu.edu.cn (B.L.); 2 Yili Engineering Research Center of Green Silicon-Based Materials, Yining 835000, China; 3 Jiangsu Key Laboratory of Artificial Functional Materials, College of Engineering and Applied Sciences, Nanjing University, Nanjing 210093, China

**Keywords:** GaN monolayer, lanthanide atoms doping, electronic and magnetic property, biaxial strain, first-principles calculations

## Abstract

The electronic and magnetic properties of lanthanide-doped GaN monolayers (Ln = La, Pr, Nd, Pm, Eu, and Gd) have been systematically investigated using density functional theory within the GGA-PBE approximation. Our results demonstrate that all Ln dopants except La introduce spin polarization and half-semiconductor behavior into the GaN monolayer. The observed magnetism primarily arises from unpaired 4f electrons, yielding magnetic moments of 2.0, 3.0, 4.0, 6.0, and 7.0 μ_B_ for Pr, Nd, Pm, Eu, and Gd, respectively. While La-, Pr-, and Gd-doped systems retain the indirect band gap characteristic of pristine GaN, an indirect-to-direct band gap transition occurs under biaxial tensile strains exceeding 2%. In contrast, Nd, Pm, and Eu doping directly induce a direct band gap without applied strain. Notably, under 6% tensile strain, the Pm- and Eu-GaN systems exhibit half-metallic and metallic properties, respectively. These tunable electronic and magnetic properties suggest that Ln doping offers a promising strategy for designing functional two-dimensional GaN-based electronic and spintronic devices.

## 1. Introduction

Following the discovery of graphene [1], numerous two-dimensional (2D) materials with unique properties have become a major research focus [2,3]. Among them, two-dimensional gallium nitride (2D GaN), first predicted through first-principles calculations [4] and subsequently synthesized via migration-enhanced encapsulated growth [5], demonstrates considerable potential for applications in spintronic and optoelectronic devices. Compared to its bulk counterpart, 2D GaN exhibits a widened bandgap due to quantum confinement effects, which enhances the operational stability of devices under high voltage [6,7]. First-principles studies further indicate that the pristine GaN monolayer adopts a graphene-like hexagonal crystal structure [8]. However, it is important to note that this monolayer possesses an indirect bandgap and exhibits non-magnetic behavior—properties inherent to its structure and elemental composition—that currently limit its direct use in spintronic applications.

First-principles calculations based on density functional theory (DFT) have become indispensable in the study of two-dimensional (2D) materials [9,10,11,12,13,14,15,16,17,18,19,20,21,22,23,24]. Numerous investigations have been conducted to modulate the physical and chemical properties of GaN monolayers, revealing that magnetism in 2D materials can be induced through various strategies such as vacancy defects, atomic adsorption, strain engineering, and doping [9,10,11,12,13,14,15]. For instance, the introduction of a neutral Ga vacancy can generate a magnetic moment of 3 μ_B_ in the GaN monolayer, while the bandgap can be tuned by varying the vacancy concentration within a specific range [9,10]. Previous work from our group demonstrated that the adsorption of atoms from groups IIIA−VIIA (excluding group VIA) can impart magnetic semiconductor or half-metallic characteristics to GaN monolayers [11]. Moreover, strain offers a straightforward and effective means to modulate material properties; applying specific levels of strain can transition the bandgap of GaN monolayer from indirect to direct [12]. Doping represents another powerful approach for tailoring the electronic and magnetic properties of 2D materials. For example, Alaal et al. investigated doping with groups IIIA−VIA elements and found that systems with group IVA and VIA dopants exhibit magnetic behavior under Ga-rich and N-rich conditions, respectively [13]. Chen et al. reported that doping with alkali or alkaline-earth metals introduces magnetic moments of 1 μ_B_ or 2 μ_B_ in GaN monolayers [14]. Transition metal (TM) doping can also induce significant magnetic moments, with Fe- and Ru-doped GaN monolayers reaching total magnetic moments of up to 5 μ_B_ [15]. Despite the high magnetic moments achievable through TM doping—attributed to the unpaired d-electrons of TM atoms—practical applications in spintronic devices remain limited due to potential quenching of d-orbital magnetic moments.

In contrast to transition metals, lanthanide (Ln) atoms exhibit magnetic properties originating from spin-parallel electrons in their highly degenerate 4f orbitals, which confer strong magnetic characteristics and remarkable resistance to orbital momentum quenching [16,17]. These attributes make Ln doping a promising strategy for designing materials that combine semiconducting behavior with robust magnetism. For example, doping blue phosphorene with lanthanide atoms has been shown to induce half-metallic or dilute magnetic semiconductor properties [16]. Furthermore, introducing Ln elements can transform nonmagnetic monolayers of ReS_2_ and HfS_2_ into ferromagnetic states [18,19]. However, the electronic and magnetic properties of Ln-doped GaN monolayers have yet to be thoroughly investigated.

Here, we systematically investigate the structural, electronic, and magnetic properties of Ln-doped GaN monolayers (where Ln = La, Pr, Nd, Pm, Eu, and Gd), along with the effects of biaxial strain. This study aims to provide theoretical guidance for the design of two-dimensional GaN-based electronic and spintronic devices.

## 2. Materials and Methods

All the calculations with spin polarization were carried out using density functional theory in AMS-BAND software (BAND 2021.104) [25]. The generalized gradient approximation (GGA) with the Perdew–Burke–Ernzerh (PBE) of functional was adopted for exchange-correlation energy together with the valence Quadruple Zeta Polarization (QZ4P) basis sets for Ln atoms and the triple-zeta polarized (TZP) basis sets for Ga and N atoms composed of slater-type and numerical orbitals without frozen core [26]. In order to model a Ln atom-doped GaN monolayer, a 4 × 4 × 1 GaN supercell was constructed. The Brillouin zones are represented by 5 × 5 × 1 and 11 × 11 × 1 *k*-point grids for the geometry optimizations and single point calculations. The energy and force convergences are set as 2 × 10^−6^ Hartree and 5 × 10^−4^ Hartree/Å, respectively. Noted that 2D periodicity is true in the AMS-BAND, namely, outside the upper and lower surfaces of the 2D materials is a semi-infinite vacuum and thus there is no need to manually add the vacuum spacing [25]. The calculated lattice constant and Ga-N bond length of GaN supercell are 12.88 and 1.86 Å and the corresponding band structure and the density of state (DOS) can be found in our previous work [10]. The strain is calculated by the expression of *ε* = (*a* − *a*_0_)/*a*_0_ where *a*_0_ and *a*, respectively, denote the lattice constants before and after the strain.

To address the well-known issue of underestimated intra-atomic Coulomb interactions among 4f electrons, the GGA+U method was employed to accurately describe the outer 4f electrons of lanthanide (Ln) atoms. The Hubbard U parameter introduces a correction to the on-site Coulomb interaction within the framework of the Hubbard Hamiltonian. We found that the choice of U value has minimal influence on the geometric structure and magnetic properties, consistent with previous reports [16,27]. Therefore, an effective U value (U_eff_) of 6 eV was selected for all calculations, in line with earlier GGA+U studies on lanthanide systems [16,28,29,30,31]. Due to the tendency of 4f electrons in Ce and Sm atoms to converge to divergent electronic and magnetic states [32,33], these elements were excluded from the current study. Charge transfer within the system was evaluated using Bader charge analysis [34].

To determine the effect of the relativity on the 4*f*-orbital of Ln atom, the crystal orbital overlap population (COOP) are calculated with the “scalar” rather than “spin-orbit” option since the spin-orbit coupling was also not considered in Ln-graphene, Ln-phosphorene, Ln-HfS_2_, and Ln-ReS_2_ systems [17,18,19,27]. Similarly, the scalar relativistic effect was considered to CsPbBr_3_ system with heavier Pb atom [35]. Moreover, the periodic energy decomposition analysis (pEDA) with the natural orbitals for chemical valency (NOCV) method are used to determine the interaction energy between Ln atoms and the remaining GaN monolayer.

## 3. Results and Discussion

### 3.1. Ln-GaN Monolayer

**Figure 1:**
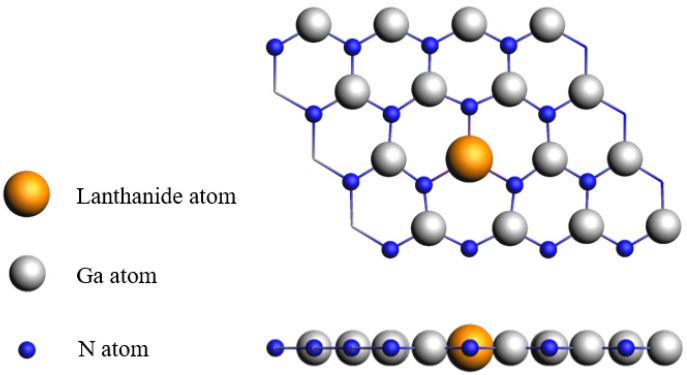
The top view and side view of 4 × 4 × 1 Ln-GaN system.

Figure 1 presents the optimized structure of the Ln-doped GaN monolayer. As shown in the side view, the monolayer retains its planar geometry after doping, indicating that the introduction of lanthanide atoms does not induce buckling—a behavior distinct from that observed in Ln-doped graphene systems [17]. The optimized structural parameters for the six considered Ln-GaN systems are summarized in Table 1. Consistent with the lanthanide contraction effect, the bond length between the dopant atom (except for Eu) and its nearest nitrogen atom decreases with increasing atomic number. The anomalous behavior of Eu, which exhibits a larger bond length, can be attributed to its relatively large atomic radius. It is worth noting, however, that the Eu-N bond length is not the longest among all Ln-N bonds, differing from trends reported in Ln-doped graphene and phosphorene systems [17,27]. This discrepancy likely stems from the fact that the valence state of Eu is +3 in the current system, as opposed to +2 in the other systems—a conclusion supported by subsequent Bader charge, crystal orbital overlap population (COOP), and magnetic moment analyses. Although the Eu-N bond length deviates from the overall trend, the lattice constants of all Ln-GaN systems decrease monotonically with increasing atomic number, suggesting significant in-plane deformation in the Eu-GaN structure. Owing to the large atomic radii of lanthanide elements, the Ln-N bond lengths range from 2.126 to 2.203 Å, which are noticeably longer than those in transition-metal-doped systems (1.840–1.990 Å) [20].

To investigate the stability of Ln-GaN system, the binding energy is calculated as follows [15,16]:

(1) $$E_{b}=E_{Ln-GaN}-E_{V}-E_{Ln}$$

where *E_Ln−GaN_*, *E_V_* and *E_Ln_* stand for the energies of Ln-GaN system, GaN monolayer with a Ga vacancy, and one isolated Ln atom, respectively. Based on this definition, the smaller the binding energy, the more stable the configuration and the corresponding results are also summarized in Table 1. The binding energies do not exhibit a monotonic trend as the number of 4*f* electrons increases, where two local minimum *E_b_* values are found in the La- and Gd-GaN systems and the maximum value appears at the Eu-GaN system. For the La atom, it has the same valence electrons as the host Ga atom. While for the Gd atom, its valence electron arrangement is 4*f*^7^5*d*^1^6*s*^2^ and the electron pairing of Gd atom with the neighbor N atom results in relatively stable 4*f*^7^ half-filled orbital state. As a result, the *E_Ln−GaN_* values of these two systems in the above equation are the two local minimum ones. On the other hand, the valence electron arrangement of Eu atom is 4*f*^7^6*s*^2^. Since the half-filled orbital state is a relatively stable electron configuration, the *E_Ln_* value of Eu atom is the smallest. Moreover, the valence state of Eu atom in the Eu-GaN system is +3 and thus the original stable 4*f*^7^6*s*^2^ configuration of Eu atoms will be destroyed, which results in the binding energy of the Eu-GaN system being the largest. As shown in Table 1, the binding energies of Ln-GaN systems are in the range of −7.700~−13.680 eV while those of Ln-graphene and Ln-phosphorene systems are −3.37~−7.13 and −4.45~−8.03 eV [17,27], indicating a strong interaction between the Ln atom and the nearest neighbor N atoms.

Based on Bader charge analysis, the calculated charge transfer values are summarized in Table 2. In the pristine GaN monolayer, each Ga atom donates 1.457e, and each N atom acquires an equivalent charge. In the Ln-doped systems, the Ln atoms lose more electrons than the host Ga atoms in the pristine monolayer due to their lower electronegativity. Consequently, the nitrogen atoms adjacent to the Ln dopants gain more electrons compared to those in the undoped GaN. Notably, the amount of charge transferred from each Ln atom to the surrounding N atoms ranges from 1.818e to 1.946e. This consistent range of electron transfer suggests a similar bonding characteristic between the Ln dopants and their neighboring N atoms [27], confirming that the valence state of the Eu atom in the Eu-GaN system is +3, rather than +2.

**Figure 2:**
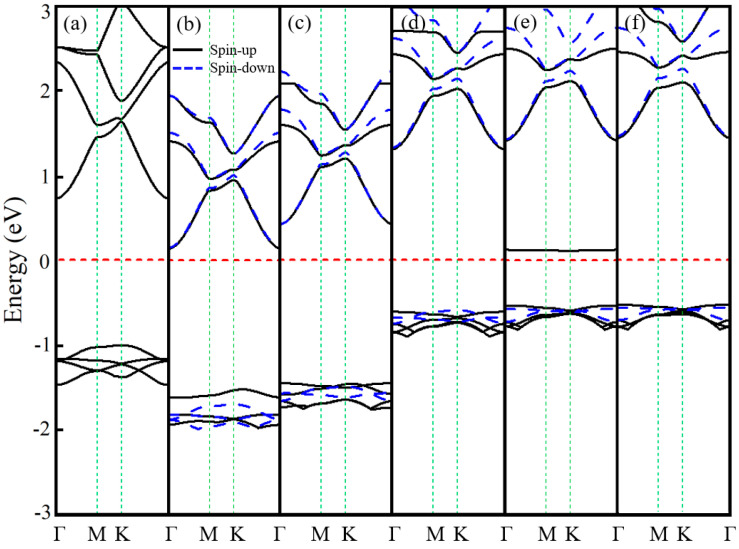
The band structures of (**a**) La- (**b**) Pr- (**c**) Nd- (**d**) Pm- (**e**) Eu-, and (**f**) Gd-GaN systems. The red horizontal dotted line represents the Fermi level.

Figure 2 displays the band structures of the six Ln-GaN systems, calculated with scalar relativity. Due to the high number of bands in the 4 × 4 supercell, only the four to six most relevant bands near the Fermi level are shown for clarity—fewer for La-GaN and more for the other systems. Compared to the pristine GaN monolayer (see Table 1), doping with La, Pr, and Nd shifts the Fermi level upward, whereas Pm, Eu, and Gd doping results in a downward shift. As summarized in Table 3, all Ln-doped systems retain semiconducting behavior but exhibit reduced band gaps relative to that of pristine GaN (2.240 eV) [10]. Specifically, the spin-up and spin-down bands of La-GaN are fully degenerate (Figure 2a), indicating the absence of spin polarization. In contrast, the other five systems exhibit pronounced spin polarization.

Notably, only the Eu-GaN system shows impurity states within the gap, resulting in the smallest band gap (0.649 eV) among all considered structures. The conduction band minimum (CBM) and valence band maximum (VBM) of La- and Pr-GaN are located at the Γ and K points, respectively, indicating an indirect band gap, while in Gd-GaN they lie at Γ and along the Γ–M path, also consistent with an indirect gap. In contrast, Nd-, Pm-, and Eu-GaN are direct-gap semiconductors, with both CBM and VBM situated at the Γ point. It is noteworthy that, except for La-GaN, all systems display majority-spin (spin-up) character at both the CBM and VBM—a signature of half-semiconductor (HSC) behavior. This property enables efficient generation and manipulation of spin-polarized currents, suggesting promising potential for spintronic applications.

**Figure 3:**
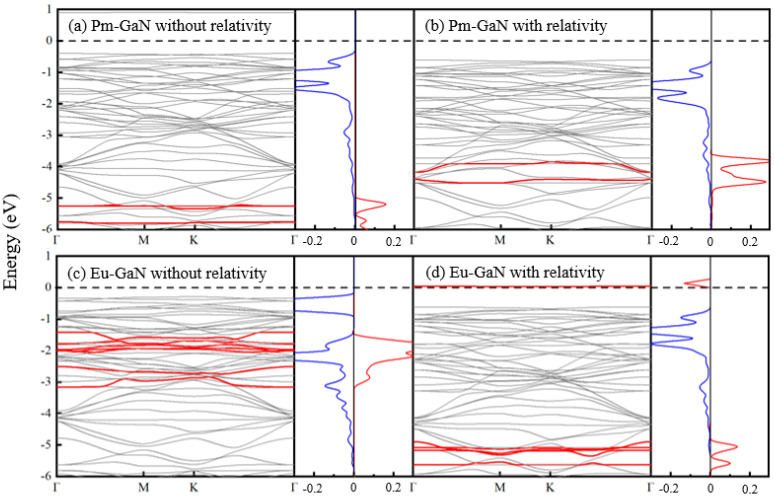
The calculated band structures and corresponding COOP results without and with the relativity, where the red and blue curves correspond to Ln-N *f*-*p* and *s*-*p* orbital interactions, respectively. The red curves represent the 4*f* orbital contribution of the Ln atom, and the *f*-*p* (red) and *s*-*p* (blue) interactions between the Ln and the nearest neighboring N atoms are listed in the COOP.

The presence of lanthanide atoms in a structure typically introduces significant relativistic effects into their orbital behaviors [23,24]. To explicitly isolate the relativistic contribution to the electronic structure, we compared band structures computed with and without scalar relativistic corrections—as implemented within the Zeroth-Order Regular Approximation (ZORA) to the Dirac equation [25]—using the same basis set. This approach allows a direct interpretation of the relativistic effects in terms of Slater-type atomic orbitals. Crystal Orbital Overlap Population (COOP) analysis, which quantifies the bonding character between pairs of orbitals or orbital shells across energy values, was employed to assess orbital interactions [23]. A positive COOP value indicates bonding states, while a negative value corresponds to antibonding states. As representative examples, the non-relativistic and scalar relativistic band structures and COOP profiles of the Pm-GaN system are presented in Figure 3a,b. Under scalar relativistic treatment, the p-orbitals of nitrogen atoms in the valence band shift slightly to lower energies, whereas the f-orbitals of the Pm atom are raised significantly in energy and exhibit enhanced spectral weight. In contrast, the f-orbitals of Eu (Figure 3c,d) shift downward in energy with reduced intensity—a behavior opposite to that of Pm. The top of the valence band in all systems is predominantly composed of antibonding states derived from σ-interactions between Ln-6s and N-3p orbitals. In contrast, bonding states formed by 4f(Ln)-3p(N) interactions appear within a relatively narrow energy range. This is consistent with the general principle that lower-lying orbitals contribute more significantly to bonding states, while higher-lying orbitals tend to dominate antibonding states [35]. The consistent bonding characteristics across all systems further support the identification of the Eu dopant in a +3 valence state. Given the similarity of the other systems to Pm-GaN, their corresponding results are not shown here.

The pEDA-NOCV method decomposes the interaction energy *E*_int_ between the two fragments—the lanthanide (Ln) atom and the GaN monolayer—into three components: *E*_int_ = *E*_elstat_ + *E*_Pauli_ + *E*_orb_ [25]. Here, *E*_elstat_ represents the electrostatic interaction between the unperturbed charge distributions of the fragments, *E*_Pauli_ denotes the Pauli repulsion arising from occupied orbital interactions, and *E*_orb_ accounts for orbital interactions involving charge transfer and polarization. As summarized in Table 4, all four energy terms exhibit a consistent variation trend: their absolute values initially decrease with increasing atomic number of the dopant, then increase for Gd doping. Consequently, two distinct minima in Eint are observed for the La- and Gd-GaN systems, while a maximum occurs in the Eu-GaN system—a trend consistent with that of the binding energy.

**Figure 4:**
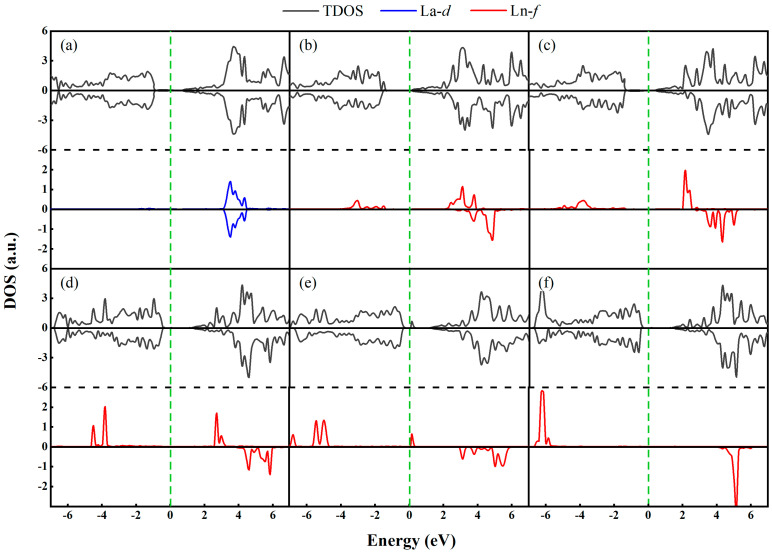
The TDOS and PDOS of (**a**) La- (**b**) Pr- (**c**) Nd- (**d**) Pm- (**e**) Eu-, and (**f**) Gd-GaN systems. The green vertical dotted line represents the Fermi level.

We now examine the total and projected density of states (TDOS and PDOS) of the Ln-GaN systems, computed at the scalar relativistic level, as shown in Figure 4. Given that the DOS near the Fermi level is dominated by the d-orbitals of La and the f-orbitals of the other Ln dopants, only the corresponding d- or f-orbital contributions are depicted in the PDOS. From Pr to Gd, the f-orbital states in the valence band shift progressively away from the Fermi level toward lower binding energies. This behavior contrasts with that observed in Ln-doped graphene and phosphorene systems, where the f-states of Eu remain close to the Fermi level due to its +2 valence state in those materials [17,27]. In the present case, however, an unoccupied impurity peak appears above the Fermi level in the Eu-GaN system, resulting in the smallest band gap (0.649 eV) among all Ln-GaN monolayers. Notably, all occupied f-orbital states exhibit spin-up polarization. The significant imbalance between occupied spin-up and spin-down states, evident in Figure 4b–f, indicates the presence of a net magnetic moment.

The contributions to the magnetic moments are summarized in Table 1. The total magnetic moment of the systems ranges from 0 to 7 μ_B_ and generally increases with the atomic number of the lanthanide dopant, correlating closely with the number of unpaired 4f electrons localized on the Ln ions. As indicated in Table 1, the magnetic moment arises primarily from the Ln atoms, with the spins on the Ln and neighboring N atoms aligned antiparallel—indicating antiferromagnetic coupling. The bonding configuration involves two electrons from the Ln 6s orbital and one electron from either the 5d or 4f orbital forming bonds with the three adjacent nitrogen atoms. The remaining spin-polarized electrons in the 4f orbitals of the Ln atoms are responsible for the observed magnetic moments. This bonding model clarifies why the total magnetic moment of the Eu-GaN system is 6 μ_B_, in contrast to the 7 μ_B_ moment reported for Eu-doped phosphorene, where the surrounding phosphorus atoms also contribute to the total magnetism [27].

### 3.2. Ln-GaN Systems at −6%~6% Biaxial Strain

Figure S1 displays the band structures of the La-GaN system under different biaxial strains. Within the compressive strain range considered, the system remains an indirect semiconductor, with the CBM located at the Γ point and the VBM at the K point. As summarized in Table 3, the band gap reaches a maximum at −4% strain, behavior consistent with that reported for pristine GaN at −5% strain [12]. In contrast, under a tensile strain of 2%, the system transitions to a direct semiconductor, as both the CBM and VBM shift to the Γ point. This differs from pristine GaN, where the indirect-to-direct transition occurs under −5% compressive strain [12]. With increasing tensile strain, both the valence and conduction bands move closer to the Fermi level, reducing the band gap to half that of the unstrained GaN monolayer at 6% strain. This tunability enhances the potential of La-GaN in electronic and optoelectronic applications. Furthermore, as shown in Figure S2, the spin-up and spin-down channels remain degenerate under all strain conditions, indicating the absence of strain-induced spin polarization. A similar trend is observed in the Pr-GaN system (Figure S3 in Supplementary Materials): the band gap is maximized at −4% strain, remains indirect under compression, and becomes direct at tensile strains ≥ 2%. Additionally, the system exhibits half-semiconductor (HSC) character within the 0% to 6% tensile strain range and at −6% compressive strain.

In the Nd-GaN system (Figure S4 in Supplementary Materials), the band gap also peaks at −4% strain, and an indirect-to-direct transition occurs at *ε* ≥ 0. HSC features are present only under tensile strains of 2%−4% and at −6% compressive strain. The complex strain dependence of the band structure in Nd-GaN arises from significant shifts in the Fermi level with applied strain.

**Figure 5:**
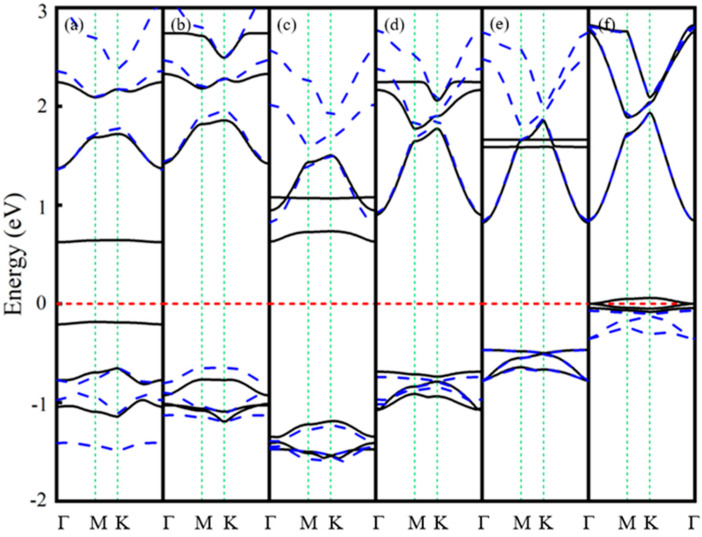
Band structures of the Pm-GaN system under biaxial strain ranging from −6% to 6% (**a**–**f**).

Similarly, the Pm-GaN system shows a maximum band gap at −4% strain and retains indirect character under compressive strain (Table 3 and Figure 5). It becomes a direct-gap semiconductor at tensile strains between 0% and 4%. At 6% tensile strain, the system exhibits half-metallic behavior, as the spin-up channel crosses the Fermi level. These impurity states near the Fermi level are primarily contributed by the Pm 4f orbitals (Figure S5 in Supplementary Materials). Additionally, the Pm-GaN system displays HSC characteristics over the strain range from −6% to 4%, except at −4%.

**Figure 6:**
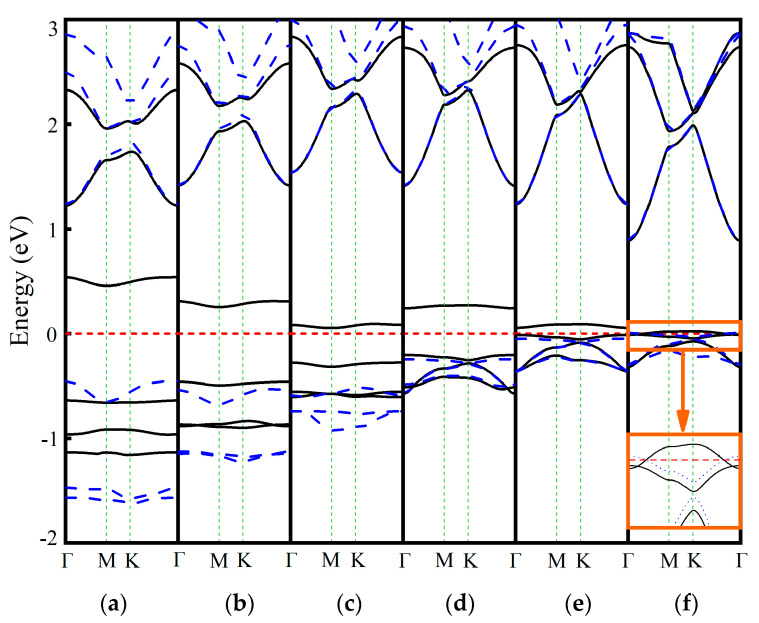
The band structures of Eu-GaN system at different strains ranging from −6% to 6% (**a**–**f**).

Figure 6 shows the calculated band structures of the Eu-GaN system under various strain. Similar to the Pm-GaN system, the band gap remains indirect under compressive strain but becomes direct within the tensile strain range of 0% to 4%. Unlike the previous four systems, the maximum band gap of the Eu-GaN system occurs at a compressive strain of −6% rather than −4%, which can be attributed to the emergence of impurity states near the Fermi level derived from the Eu 4f orbitals (Figure S6 in Supplementary Materials). At a tensile strain of 6%, the system exhibits metallic behavior, as both spin-up and spin-down channels cross the Fermi level. Furthermore, the Eu-GaN system displays HSC characteristics within the strain range of −4% to 4%.

Similar to La- and Pr-GaN systems, the band gap of Gd-GaN system also has a maximum value at −4% strain and remains indirect under compressive strain while it becomes direct from 2% tensile strain, as shown in Figure S7. Moreover, this system shows HSC feature in the whole tensile strain range.

## 4. Conclusions

In summary, the structural, electronic, and magnetic properties of Ln-doped GaN monolayers were investigated using the GGA+U method. The results indicate that the chemical stability of the Ln-GaN systems, as well as the interaction between the lanthanide dopants and the GaN monolayer, decrease from La to Eu but increase significantly for the Gd-doped system. The pristine indirect band gap of GaN is preserved upon doping with La, Pr, and Gd, while doping with Nd, Pm, and Eu results in a direct band gap. Furthermore, all Ln dopants except La introduce spin polarization and half-semiconductor characteristics. The computed magnetic moments—2.0, 3.0, 4.0, 6.0, and 7.0 μ_B_ for Pr through Gd—correlate with the number of unpaired 4f electrons, and confirm a +3 valence state for Eu in the Eu-GaN system, in contrast to the +2 state reported in other substrates. Under compressive strain, all Ln-GaN systems maintain an indirect band gap, whereas under tensile strain they generally become direct-gap semiconductors, except for the Pm- and Eu-GaN systems, which exhibit half-metallic and metallic behavior, respectively, at 6% tensile strain. These findings demonstrate that lanthanide doping combined with biaxial strain can effectively diversify the electronic properties and functionalities of GaN monolayers, offering valuable theoretical insights for the design of GaN-based electronic and spintronic devices.

While this work demonstrates the great potential of lanthanide doping in tuning the properties of GaN monolayers, it also highlights the associated computational challenges. The complex electronic structure of Ln atoms, particularly the strongly correlated 4f electrons and their large atomic radii, necessitates sophisticated methods like GGA+U and large supercells, making systematic investigations computationally demanding and time-consuming. However, overcoming these challenges opens up immense opportunities for future research. Firstly, the predicted half-semiconducting and strain-tunable half-metallic properties make Ln-doped GaN a prime candidate for next-generation spintronic devices, such as spin filters and spin transistors. Secondly, exploring a wider range of lanthanide elements could unveil new magnetic phenomena and quantum states. Thirdly, experimental synthesis of these systems, though challenging, would be a groundbreaking step towards practical applications. Techniques like molecular beam epitaxy (MBE) or advanced doping processes during the growth of 2D GaN could be explored. Finally, the interplay between strain, doping, and magnetic coupling revealed in this work suggests that external fields (electric or magnetic) could provide additional control knobs, promising for designing multifunctional devices. We hope this work will stimulate further theoretical and experimental efforts in this fascinating field.

## Figures and Tables

**Table 1:** The optimized lattice constant (*a*), bond length between the dopant and the nearest N atom (*l_Ln-N_*), binding energy (*E_b_*), and magnetic contributions of Ln and its nearest neighboring N and Ga atoms.

Dopant	*a* (Å)	*l_Ln-N_* (Å)	Fermi Level (eV)	*E_b_* (eV)	M_tot_ (μ_B_)	M_Ln_ (μ_B_)	M_N_ (μ_B_)
None	12.880	1.865	−4.370	/	0	0	0
La	13.070	2.203	−3.882	−11.450	0	0	0
Pr	13.046	2.154	−3.312	−9.773	2.0	2.017	−0.008
Nd	13.042	2.152	−3.582	−9.296	3.0	3.007	−0.009
Pm	13.037	2.147	−4.484	−9.477	4.0	4.028	−0.012
Eu	13.036	2.153	−4.608	−7.700	6.0	6.051	−0.017
Gd	13.028	2.126	−4.631	−13.680	7.0	7.029	−0.013

**Table 2:** In the case of atomic charge gain and loss. N1 and Ga1, respectively, denote the nearest neighboring N and Ga atoms to the Ln atom, while N2 is the next nearest neighboring N atom. A negative value for the charge transfer indicates electron donation from the dopant to the GaN plane, while a positive value signifies electron transfer in the opposite direction.

Dopant	Ln	N1	Ga1	N2
None	/	1.457	−1.457	/
La	−1.946	1.561	−1.433	1.457
Pr	−1.820	1.526	−1.440	1.457
Nd	−1.818	1.534	−1.444	1.455
Pm	−1.828	1.536	−1.442	1.458
Eu	−1.870	1.566	−1.450	1.457
Gd	−1.861	1.551	−1.442	1.458

**Table 3:** The band gaps of Ln-GaN systems at −6%~6% strain range.

Dopant	−6%	−4%	−2%	0	2%	4%	6%
La	1.881	1.895	1.839	1.728	1.559	1.213	0.860
Pr	1.694	1.893	1.856	1.662	1.609	1.248	0.881
Nd	1.770	2.034	1.930	1.871	1.678	1.284	0.861
Pm	0.808	2.070	1.812	1.892	1.589	1.291	/
Eu	0.840	0.701	0.317	0.649	0.445	0.069	/
Gd	2.090	2.144	2.117	1.972	1.640	1.281	0.909

**Table 4:** The pEDA-NOCV results.

Dopant	*E*_Pauli_ (eV)	*E*_elstat_ (eV)	*E*_orb_ (eV)	*E*_int_ (eV)
La	32.258	−21.639	−23.743	−13.124
Pr	29.557	−20.334	−21.188	−11.965
Nd	28.984	−20.199	−19.992	−11.208
Pm	28.478	−19.858	−19.371	−10.751
Eu	26.670	−18.653	−17.274	−9.257
Gd	29.930	−20.424	−22.308	−12.802

## Data Availability

The data presented in this study are contained within the article.

## Supplementary Materials

The following supporting information can be downloaded at: https://www.mdpi.com/article/10.3390/nano15171331/s1. Figure S1: The band structures of La-GaN system at the strain from −6% (a) to 6% (f); Figure S2: The TDOS and PDOS of La-GaN system at the strain from −6% (a) to 6% (f). The green vertical dotted line located at 0 eV represents the Fermi level; Figure S3: The band structures of Pr-GaN system at the strain from −6% (a) to 6% (f); Figure S4: The band structures of Nd-GaN system at the strain from −6% (a) to 6% (f); Figure S5: The TDOS and PDOS of Pm-GaN system at the strain from −6% (a) to 6% (f); Figure S6: The TDOS and PDOS of Eu-GaN system at the strain from −6% (a) to 6% (f); Figure S7: The band structures of Gd-GaN system at the strain from −6% (a) to 6% (f).
