# Supplementary material for: Tuning of the Electronic and Magnetic Properties of GaN Monolayers via Doping with Lanthanide Atoms and by Applying Biaxial Strain
Source: Nanomaterials (Basel). 2025 Aug 29;15(17):1331. doi: 10.3390/nano15171331 (PMC12430650; doi:10.3390/nano15171331)
Supplement: Supplementary file 1 [file nanomaterials-15-01331-s001.zip › nanomaterials-3813732-supplementary.pdf]

nanomaterials

MDPI

Supplementary Materials

Tuning of the Electronic and Magnetic Properties of GaN Monolayers via Doping with Lanthanide Atoms and by Applying Biaxial Strain

Xue Wen [1,2], Bocheng Lei [1,2], Lili Zhang *[1,2] and Haiming Lu *[1,3]

1 Xinjiang Laboratory of Phase Transitions and Microstructures in Condensed Matters, College of Physical Science and Technology, Yili Normal University, Yining 835000, China; 19899036782wx@sina.com (X.W.); leibocheng@ylnu.edu.cn (B.L.)
2 Yili Engineering Research Center of Green Silicon-based Materials, Yining 83500, China.
3 College of Engineering and Applied Sciences, Jiangsu Key Laboratory of Artificial Functional Materials, Nanjing University, Nanjing 210093, China.
* Correspondence: zhanglili@ylnu.edu.cn (L.Z.); haimlu@nju.edu.cn (H.L.)

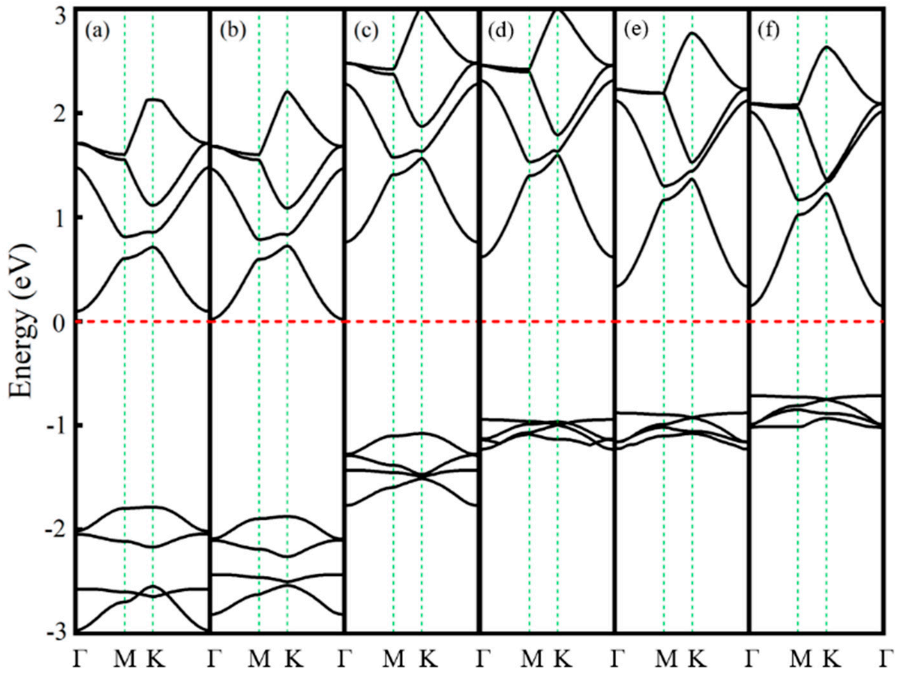

Figure S1. The band structures of La-GaN system at the strain from −6% (**a**) to 6% (**f**).

 https://doi.org/10.3390/nano15171331

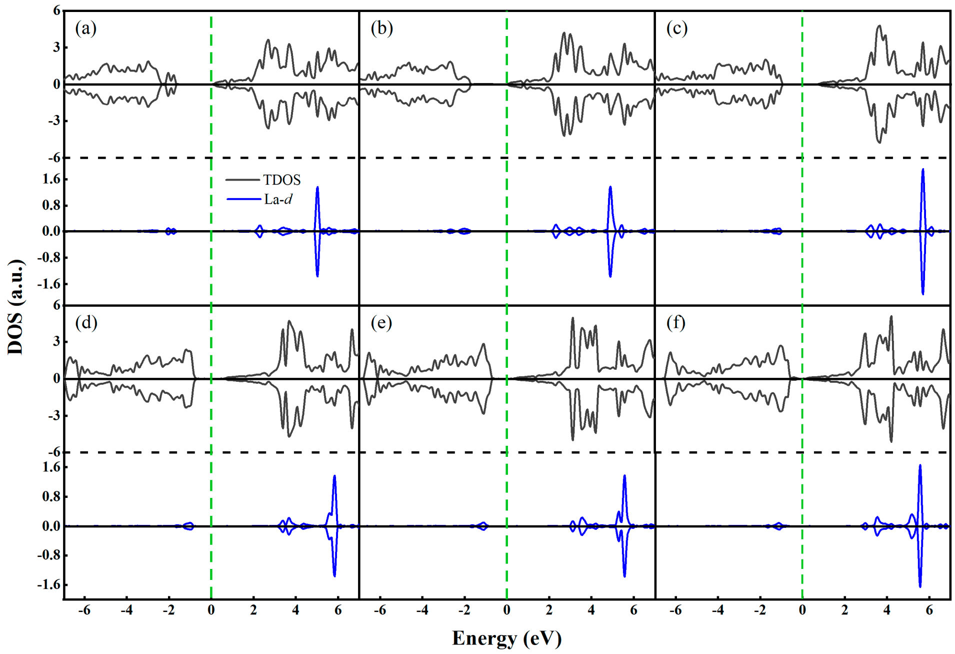

Figure S2. The TDOS and PDOS of La-GaN system at the strain from −6% (**a**) to 6% (**f**). The green vertical dotted line located at 0 eV represents the Fermi level.

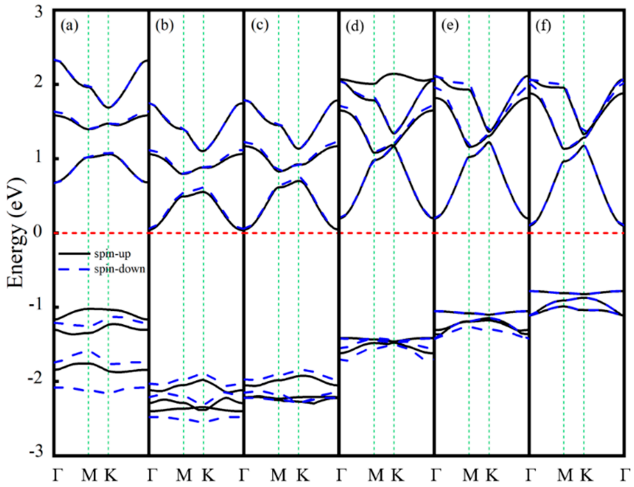

Figure S3. The band structures of Pr-GaN system at the strain from −6% (**a**) to 6% (**f**).

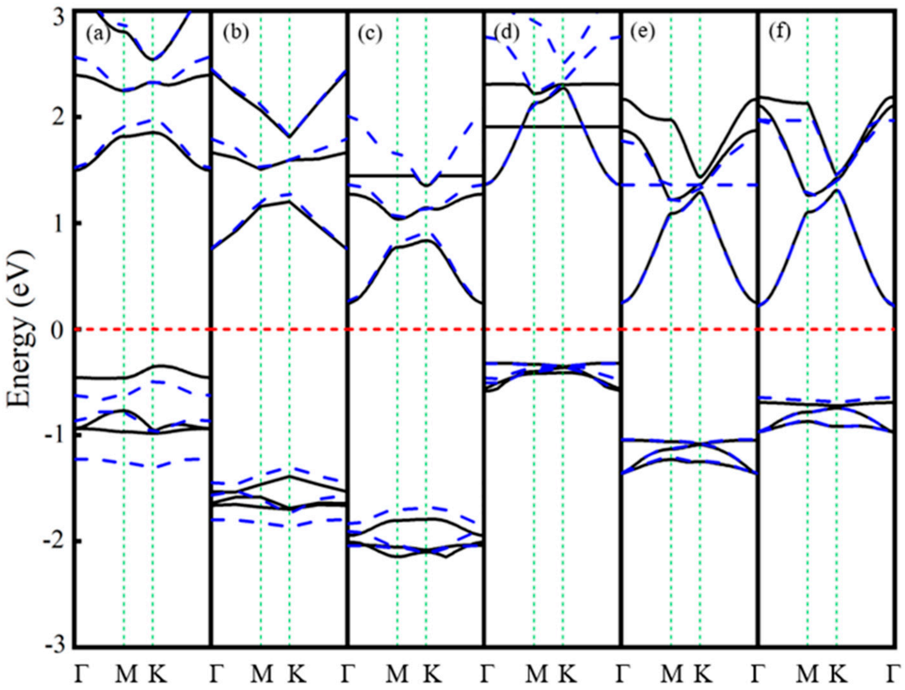

Figure S4. The band structures of Nd-GaN system at the strain from −6% (**a**) to 6% (**f**).

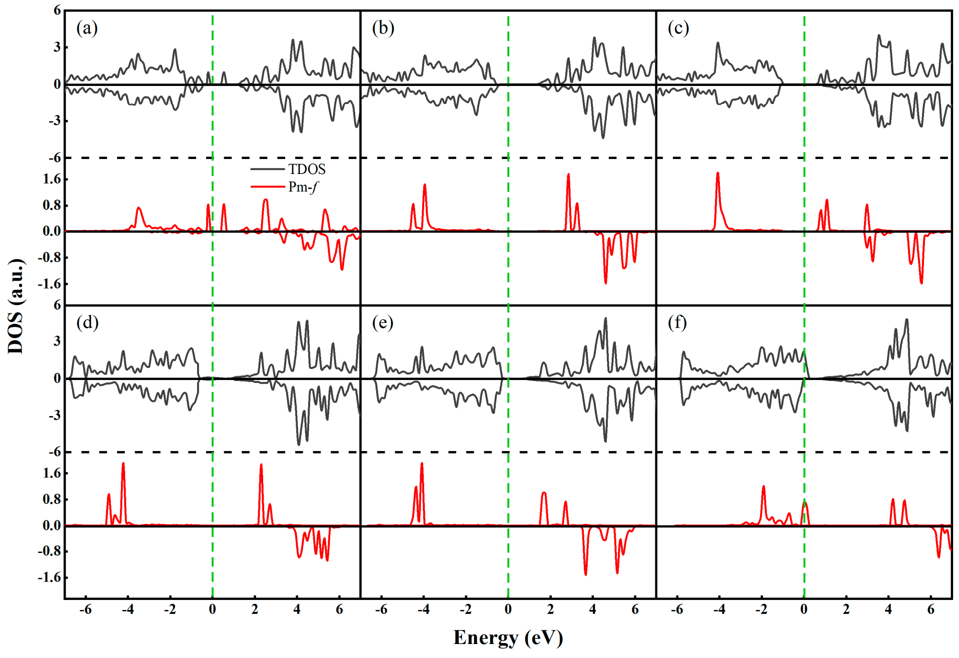

Figure S5. The TDOS and PDOS of Pm-GaN system at the strain from −6% (**a**) to 6% (**f**).

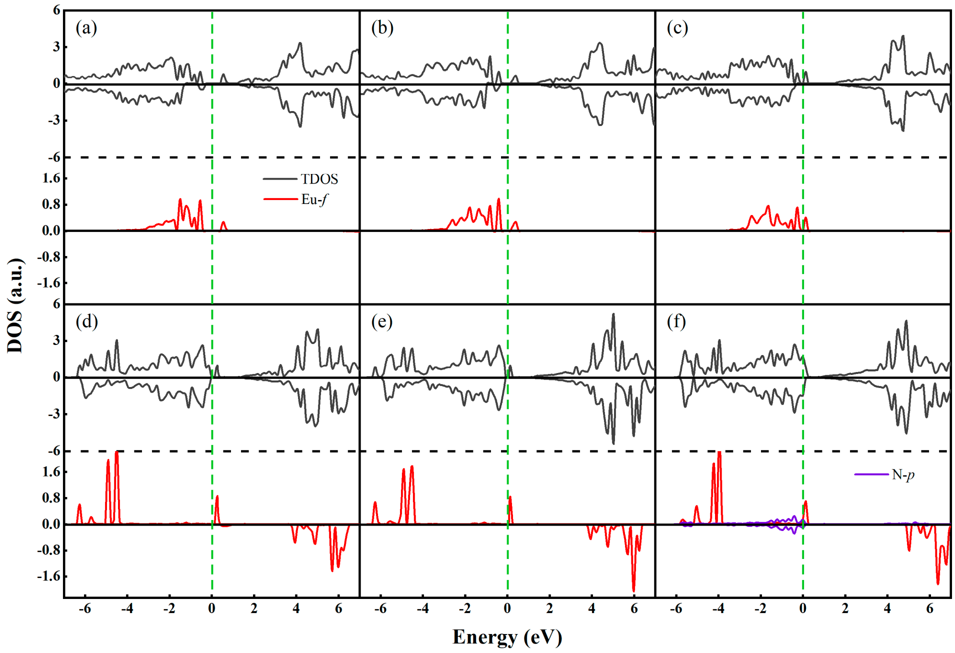

Figure S6. The TDOS and PDOS of Eu-GaN system at the strain from −6% (**a**) to 6% (**f**).

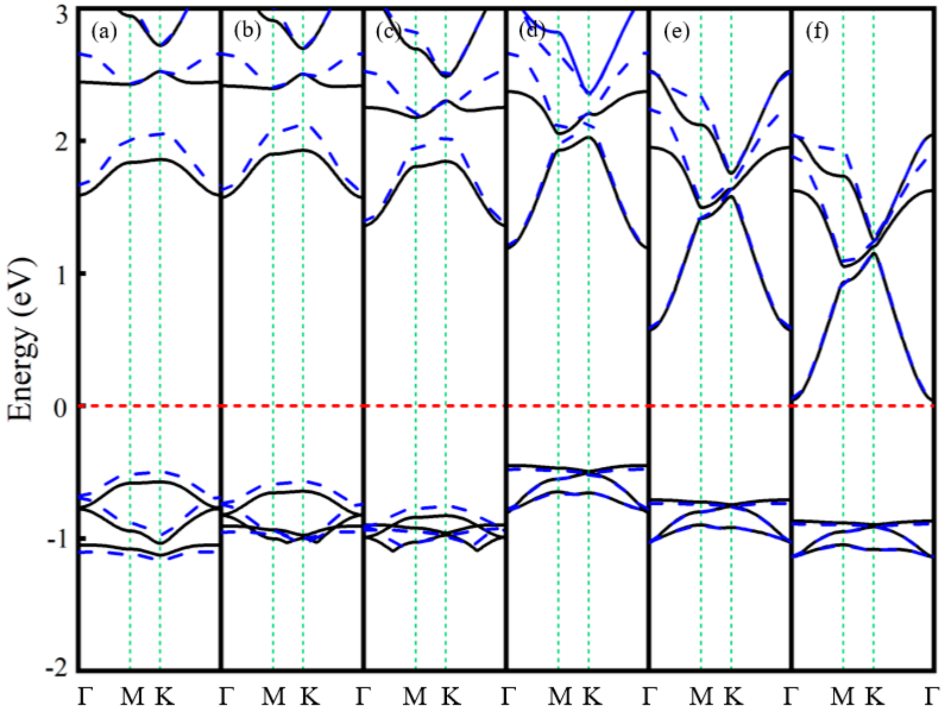

Figure S7. The band structures of Gd-GaN system at the strain from −6% (**a**) to 6% (**f**).

Disclaimer/Publisher's Note: The statements, opinions and data contained in all publications are solely those of the individual author(s) and contributor(s) and not of MDPI and/or the editor(s). MDPI and/or the editor(s) disclaim responsibility for any injury to people or property resulting from any ideas, methods, instructions or products referred to in the content.
